# Oxidative Stress Predicts Post-Surgery Complications in Gastrointestinal Cancer Patients

**DOI:** 10.1245/s10434-022-11412-8

**Published:** 2022-02-17

**Authors:** M. Leimkühler, A. R. Bourgonje, H. van Goor, M. J. E. Campmans-Kuijpers, G. H. de Bock, B. L. van Leeuwen

**Affiliations:** 1 Department of Surgery, University of Groningen, University Medical Center Groningen, Groningen, The Netherlands; 2 Department of Gastroenterology and Hepatology, University of Groningen, University Medical Center Groningen, Groningen, The Netherlands; 3 Department of Pathology and Medical Biology, University of Groningen, University Medical Center Groningen, Groningen, The Netherlands; 4 Department of Epidemiology, University of Groningen, University Medical Center Groningen, Groningen, The Netherlands

**Keywords:** Oxidative stress, Thiols, Gastrointestinal cancer, Postoperative complications

## Abstract

**Introduction:**

An excessive perioperative inflammatory reaction can lead to more postoperative complications in patients treated for gastrointestinal cancers. It has been suggested that this inflammatory reaction leads to oxidative stress. The most important nonenzymatic antioxidants are serum free thiols. The purpose of this study was to evaluate whether high preoperative serum free thiol levels are associated with short-term clinical outcomes.

**Methods:**

Blood samples were drawn before, at the end of, and 1 and 2 days after surgery of a consecutive series of patients with gastrointestinal cancer. Serum free thiols were detected using a colorimetric detection method using Ellman’s reagent. Short-term clinical outcomes were defined as 30-day complications (Clavien-Dindo ≥2) and length of hospital stay. Logistic regression was applied to examine the association between serum free thiol levels and short-term patient outcomes.

**Results:**

Eighty-one patients surgically treated for gastrointestinal cancer were included in the study. Median age was 68 (range 26–87) years, and 28% were female. Patients in the lowest tertile of preoperative serum free thiols had a threefold higher risk to develop postoperative complications (odds ratio [OR]: 3.4; 95% confidence interval [CI]:1.1–10.7) and a fourfold higher risk to have an increased length of stay in the hospital (OR 4.0; 95% CI 1.3–12.9) compared with patients in the highest tertile.

**Conclusions:**

Patients with lower preoperative serum free thiol levels, indicating a decrease in extracellular antioxidant capacity and therefore an increase in systemic oxidative stress, are more likely to develop postoperative complications and show a longer in hospital stay than patients with higher serum free thiol levels.

**Supplementary Information:**

The online version contains supplementary material available at 10.1245/s10434-022-11412-8.

Oncological surgery forms the cornerstone of treatment in patients with gastrointestinal cancer. However, 14–34% of these patients experience postoperative complications, leading to disability and even death in up to 25% of frail older patients within the first year after surgery.^1–3^ Moreover, postoperative complications can lead to a prolonged hospital stay, higher readmission rate, and therefore higher healthcare costs.^4,5^ The identification of risk factors for postoperative complications can help with treatment selection for a patient and offers possibilities to reduce complications. Previous studies have shown that preoperative factors, such as comorbidities, sarcopenia, and obesity, increase the risk of complications.^2,6,7^ Furthermore, elevated levels of inflammatory markers, such as IL-6, IL-8, and IL-10, before surgery have been associated with postoperative complications.^8–11^

Excessive systemic inflammation is intimately associated with higher levels of oxidative stress, which is defined as an imbalance between the production of reactive species and the availability of antioxidant substances.^12^ Due to this association, systemic markers of oxidative stress might serve as new prognostic biomarkers for postoperative complications. Oxidative stress is defined by a reduction-oxidation (redox) imbalance, which is characterized by an excessive production of reactive species, while antioxidant availability is decreased.^13–16^ A robust and powerful read-out of an individual redox status is the measurement of a patients’ serum or plasma free thiols (R-SH, sulfhydryl compounds).^15,17–19^ These constitute the most important nonenzymatic antioxidants, because they represent an intricate and dynamic redox regulation system, acting as major scavengers of reactive species as well as serving as multimodal redox relays (*redox switche*s) by kinetically controlling redox exchange reactions within the *reactive species interactome* (RSI), culminating into chemical modifications with both short-term and longer-term biological adaptations.^20,21^ Free thiols can easily be measured in patients’ serum or plasma using established colorimetric detection methods.^22,23^ Free thiols have a potent antioxidant capacity, due to their sulfhydryl group (-SH), which enables them to form reversible disulfide bonds with reactive species (RS), thus balancing the effect of oxidative stress.^14–17,24^ A lower antioxidant capacity, or higher levels of oxidative stress, is characterized by reduced levels of serum free thiols.^25^

Several studies described lower serum free thiol levels in patients with cancer compared with healthy people.^15,26–30^ Lower serum thiol levels have been associated with lower survival rates in some studies.^15,31^ Furthermore, it has been shown that patients experience a decrease in serum thiol levels after a prostate biopsy.^16^ An oncological surgery might deplete the antioxidant capacity of the patients even more, as it is more extensive than a biopsy. Additionally, a patient with an already higher level of oxidative stress might be more prone to complications after oncological surgery. Therefore, the purpose of this study was to evaluate the association between surgery and systemic redox status of patients with gastrointestinal cancers and the association between serum free thiol concentrations and short-term patient outcomes measured by the occurrence of 30-day complications and the length of hospital stay.

## Methods

### Study Population

Data for this observational cohort study were prospectively collected in the setting of an observational cohort study conducted in the University Medical Center Groningen (UMCG) as described elsewhere.^11,32–34^ Patients were included from April 2014 till October 2016 following a previously determined study protocol. Eligible for inclusions were all patients, aged 18 years and older, who received elective oncological surgery in the UMCG, regardless of the primary tumor. Excluded were patients with a Karnofsky Performance Score <80 or the inability to provide written, informed consent. In our study, we included a subcohort of all patients with a diagnosis of gastrointestinal cancer that were included in the observational cohort. All patients signed a written, informed consent for the use of their data and plasma samples. The study (trial number NL 45602.042.14) was approved by the Institutional Review Board (IRB) of the UMCG (in Dutch: “Medisch Ethische Toetsingscommissie, METc”) and conducted in accordance with the principles of the Declaration of Helsinki (2013).

### Data Collection

Demographics, such as age, sex and body mass index (BMI), Charlson comorbidity, and ASA score were prospectively registered for all patients during the study.^35,36^ In addition, the type of tumor and its treatment has been registered. Treatment registration included the type of surgery and whether patients received neoadjuvant chemotherapy. Complications within 30 days after surgery according to Clavien-Dindo Classification (CDC) and length of hospital stay were prospectively registered.^37^

### Blood Sampling and Biochemical Analysis

Blood samples were drawn in the morning before surgery, at skin closure, and on the first and second postoperative day to illustrate the changes of biomarkers in time. Blood samples were handled as described previously.^33^ All blood samples were directly centrifuged at 2600×*g* for 10 min, after which they were stored at a minimum of −80 °C. All blood was analyzed for C-reactive protein (CRP) (lower limit of detection [LLD]: 0.001 μg/ml), interleukin-1β (IL-1β) (LLD: 1.27 pg/ml), IL-6 (LLD: 0.01 pg/ml), IL-10 (LLD: 3,28 pg/ml), IL-12 (LLD: 5.07 pg/ml), and tumor necrosis factor-alpha (TNF-α) (LLD: 6.49 pg/ml). Haemoscan^®^ (Groningen) analyzed all samples in batches (measured in singular) using sandwich enzyme-linked immunosorbent assays (ELISA) technique for interleukins, developed by BioLegend (San Diego, CA) and high sensitivity CRP ELISA (Dakopatts, Glostrup, Denmark) for CRP.

### Measurement of Serum Free Thiols

As a read-out for the systemic redox-status of the patient, free thiol levels were determined. Analysis of the serum samples was executed within the UMCG according to earlier published work.^38^ Dilution of serum samples was done using 0.1 M Tris buffer (pH 8.2). The background absorption was measured using a Variskan microplate reader, together with a reference measurement at 630 nm. After that 20 μ of 1.9 nM 5,5’-dithio-bis (2-nitrobenzoic acid) (DTNB, Ellman’s Reagent, CAS-number 69-78-3, Sigma Aldrich Corporation, St. Louis, MO) in 0.1 M of phosphate buffer (pH 7.0) was added to the samples. The samples were incubated for 20 minutes at room temperature, after which absorbance was measured. Parallel measurement of an L-cysteine CAS-number 52-90-4, Fluka Biochemika, Buchs, Switzerland) calibration curve (concentration range from 15.6 M to 1000 M) in 0.1 M Tris/10 mM EDTA (pH 8.2) determined the final concentrations of free thiol groups in the serum.

### Endpoints

The primary endpoint was the occurrence of postoperative complications of grade 2 or above according to the Clavien-Dindo classification within 30 days after surgery. The secondary outcome was the length of hospital stay. Preoperative serum free thiol levels were used as main predictor.

### Statistical Analysis

Clinical characteristics and baseline demographics were presented as mean plus standard deviation (SD), or in case of nonnormal distributions as median plus interquartile range (IQR) or as proportions *n* with their percentages (%). Normality testing was performed using Shapiro-Wilk tests. Serum free thiol levels were divided into tertiles and incorporated as categorical predictor in logistic regression analysis. Univariable logistic regression analyses were performed to examine the associations between predictor variables and the occurrence of postoperative complications and the length of hospital stay. Results were expressed as odds ratios (ORs) with corresponding 95% confidence intervals (CIs). Following that, multivariable logistic regression analyses were performed to examine the association between predictor variables taking the relevant covariates into account. A linear-regression analysis was performed to estimate whether the postoperative change in serum free thiol levels was influenced by operative blood loss and length of anesthesia. Statistical analyses were performed using IBM SPSS Statistics software package (version 23.0) for Windows (SPSS Inc. Chicago, IL). Data visualization was performed using GraphPad Prism version 7.02 (GraphPad software, San Diego, CA). Two-tailed *p*-values ≤ 0.05 were considered statistically significant.

## Results

### Characteristics of Patients

Of the 143 patients who enrolled in the initial study, we included 81 patients with a diagnosis of gastrointestinal cancer. Baseline serum free thiol levels were available of 75 of the 81 patients. Of these patients, 49 (60.5%) were diagnosed with lower gastrointestinal cancer, including colon cancer, rectal cancer, and appendiceal cancer, whereas 32 (39.5%) patients were diagnosed with upper gastrointestinal cancer, including esophageal cancer, gastric cancer, and small intestinal cancer. Patients with the lowest level of baseline serum free thiol levels more often received neoadjuvant chemotherapy and were more likely to have a stage 3–4 tumor. Baseline demographics and clinical characteristics are presented in Table 1.

**Table 1:** Patient characteristics for patients with baseline thiol levels above and below the median

Characteristic	Free thiol levels first tertile (172.7–252.3), *n* = 25	Free thiol levels second tertile (252.4–295.7), *n* = 24	Free thiol levels third tertile (295.8–316.6), *n* = 26
Age (year)	68 (61.5–73)	70.5 (53-86)	67.0 (26-87)
Female, *n* (%)	7 (28.0)	7 (29.2)	8 (30.8)
Body mass index (kg/m^2^)	27.4 (±3.2)	26.9 (±3.4)	25.9 (±2.6)
Charlson comorbidity index, *n* (%)			
2-4	9 (36.0)	8 (33.3)	12 (46.2)
5-9	16 (64)	16 (66.6)	14 (53.8)
ASA, *n* (%)			
I-II	17 (68)	17 (70.8)	21 (80.8)
III-IV	8 (32)	7 (29.2)	5 (19.2)
Primary tumor, *n* (%)			
Upper GI	14 (56.0)	5 (20.8)	9 (34.6)
Lower GI	11 (44.0)	19 (79.2)	17 (65.4)
Neoadjuvant chemotherapy, *n* (%)	16 (64.0)	13 (54.1)	5 (19.2)
Tumor stadium, *n* (%)			
I–II	2 (8.0)	5 (20.8)	5 (19.2)
III–IV	19 (76.0)	15 (62.5)	12 (46.2)
Unknown	4 (16.0)	4 (16.7)	9 (34.6)
Type of surgery			
Laparoscopy	6 (24.0)	3 (12.5)	8 (30.8)
Laparotomy	19 (76.0)	21 (87.5)	18 (69.2)

*GI* gastrointestinal tract; *Upper GI* esophagus, gastric, small intestines; *Lower GI* colon, rectum, appendix

### Postoperative Outcomes

Within 30 days, 34 (42.0%) patients developed complications of Clavien-Dindo Grade 2 or above (Table 2). Two patients experienced a re-admission. Median length of hospital stay was 10 (range 7–15) days.

**Table 2:** Postoperative outcomes

Length of stay in days, median (25th, 75th percentile)	10 (7–15)
Complications following Clavien-Dindo, *n* (%)	
None	33 (44.0)
1	8 (10.7)
2	21 (28.0)
3a	6 (8.0)
3b	3 (4.0)
4a	4 (5.3)

### Changes in Biomarkers

**Fig. 1.**
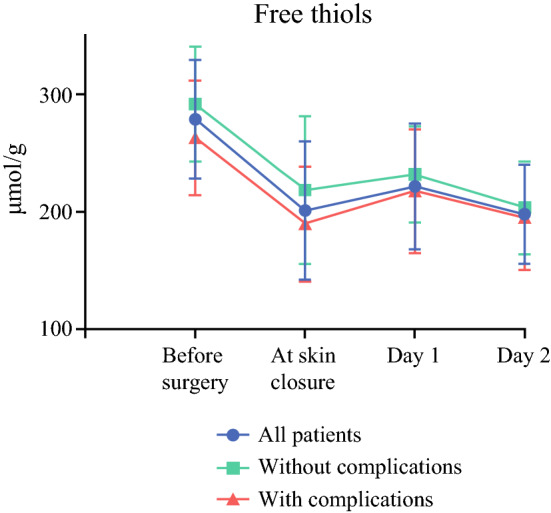
Development of free thiol levels over time

Serum free thiol levels were normally distributed, whereas the other biomarkers showed no normal distribution. Postoperative changes in serum concentrations of free thiols are depicted in Fig. 1. Free thiols decreased directly after surgery. Changes in serum concentrations of other inflammatory markers can be found in supplementary Table 1. A linear regression analysis showed that free thiol levels decreased significantly more in patients with more blood loss (−0.04 for every ml, *p* < 0.01) and in patients with a longer duration of anesthesia (−0.09 for every min of anesthesia, *p* < 0.01).

### Association of Biomarkers and Short-Term Outcomes

#### Postoperative Complications

To ascertain the effect of free thiols and other predictors on postoperative complications with a CDC ≥ 2, a logistic regression analysis was performed. Results are presented in Table 3. Patients in the lowest tertile of preoperative free thiol levels had an odds ratio of 3.4 (95% CI 1.1–10.7, *p* = 0.04), and patients in the second tertile had an odds ratio of 1.9 (95% CI 0.6–6.1, *p* = 0.28) for developing postoperative complications compared with patients in the highest tertile. Multivariable analysis showed an odds ratio of 4.6 (95% CI 0.94–22.1, *p* = 0.06) and 2.0 (95% CI 0.4–9.2, *p* = 0.38) for patients in the lowest and second tertile, respectively, compared with patients in the highest tertile of preoperative free thiols (Table 4).

**Table 3:** Univariate logistic regression analysis for characteristics at baseline and complications grade 2 or more within 30 days after surgery

	OR (95% CI)	*p* value
Free thiol		
First tertile (172.7–252.3)	3.4 (1.1–10.7)	0.04
Second tertile (252.4–295.7)	1.9 (0.6–6.1)	0.28
Third tertile (295.8–316.6)	1	
Gender		
Female	0.79 (0.3–2.1)	0.64
Male	1	
Age (year)		
≤70	0.96 (0.4–2.4)	0.96
>70	1	
BMI		
Normal BMI (<25)	1	
Overweight (25-30)	1.46 (0.4 – 6.4)	0.62
Obese (>30)	2.40 (0.8 – 7.4)	0.12
Comorbidity		
≤ 4	1	
> 4	1.23 (0.5–3.1)	0.66
Tumor stadium		
I - II	1	
III - IV	1.5 (0.4–5.1)	0.54
Neoadjuvant chemotherapy		
No	1	
Yes	1.7 (0.7–4.3)	0.24
Type of surgery		
Laparascopy	1	
Laparatomy	1.1 (0.4–3.4)	0.91

*OR* odds ratio; *CI* confidence interval; *BMI* body mass index

**Table 4:** Multivariate logistic regression analysis for characteristics at baseline and complications grade 2 or more within 30 days after surgery

	OR (95% CI)	*p* value
Free thiol		
First tertile (172.7–252.3)	4.6 (0.94–22.1)	0.06
Second tertile (252.4–295.7)	2.0 (0.4–9.2)	0.38
Third tertile (295.8–316.6)	1	
Gender		
Female	0.8 (0.2–3.3)	0.76
Age (year)		
>70	1.6 (0.4–5.9)	0.46
BMI	1.1 (0.4–3.0 )	0.82
Comorbidity		
≤4	0.84 (0.2–3.1)	0.80
Tumor stadium	1.2 (0.3–5.2)	0.81
Neoadjuvant chemotherapy		
Yes	1.6 (0.4–6.8)	0.50
Type of surgery		
Laparatomy	1.8 (0.4–7.6)	0.41

#### Length of Stay

Logistic regression showed that patients in the lowest tertile of preoperative free thiol levels had a fourfold risk (OR: 4.0; 95% CI 1.3-12.9, *p* = 0.01) to have a length of stay above the median compared with patients in the highest tertile (supplementary Table 2).

## Discussion

This observational study shows that patients with relatively lower preoperative serum free thiol levels were more likely to develop complications postoperatively and have a fourfold higher odds to have a longer in-hospital stay than patients with higher serum free thiol levels. Furthermore, it is demonstrated that serum-free thiol levels decreased directly after surgery without short-term recovery.

Until this date, these associations have not yet been described in the context of gastrointestinal cancer. However, a relationship between lower total thiol levels and lower Karnofsky performance scores, which are known to be related to patient functioning, has been shown.^39^ In addition, a study by Topuz *et al*. showed that patients with lower free thiol levels are more prone to develop chemotherapy-induced cardiac toxicity.^24^

Although there is no literature on the association of serum free thiol levels and complications, an association between lower serum thiol levels and lower survival rates has been described previously. In a study of 3361 patients with colorectal cancer, patients with lower serum total thiols had a higher mortality rate both due to colorectal cancer-related deaths as well as death due to other causes compared with patients with higher levels of serum total thiols.^40^ The association of lower serum free thiol levels and a decreased survival also has been described in non-small cell lung cancer patients, hepatic cancer, and oral squamous carcinoma.^15,31,41,42^ Also outside of oncology, a lower survival rate and a higher likelihood of graft failure has been described in renal transplant recipients with lower serum free thiols.^43^ Furthermore, a higher all-cause mortality has been described for people with lower free thiol levels in the general population.^44^ It can be hypothesized that these increased mortality rates are caused by postoperative complications. First, complications can be the direct cause of death of a patient. Second, it has been described that patients with more complications have a lower survival rates than patients without complications.^45–47^ Although the reason for this has not yet been specified, complications may cause a prolonged or incomplete recovery. In addition, it might be impossible to administer necessary adjuvant treatments to a patient with complications. Why some patients have lower thiol levels before surgery remains unclear. This might be due to the presence of the tumor itself, which has been described in colorectal cancer as well as other nongastrointestinal cancers.^27–29,41^ However, genetic factors or dietary influences might also play a role, but none of these studies has data on thiol levels before the patients diagnosis of cancer, leaving the cause of lower thiol levels unclear. However, we do know that some lifestyle factors influence free thiol levels. Smoking and a higher BMI both have a negative correlation with serum free thiol levels, whereas exercise is positively correlated with serum free thiol levels.^44,48–50^

In our study, we observed that serum free thiol levels decreased directly after surgery, which might reflect the patients’ inflammatory reaction leading to oxidative stress as the result of an imbalance between reactive species and antioxidants. Furthermore, our study showed that serum free thiol levels decreased significantly more in patients experiencing more blood loss and in patients with a longer time of anesthesia, reflecting the surgical stress of the patient. Our results are directly in line with earlier findings. One study among 22 men described a decrease in serum free thiols directly after prostate biopsy, which is most likely due to a spike in oxidative stress directly after the procedure.^16^ Another study described 68 patients who received either elective laparoscopic cholecystectomy or open inguinal femoral hernia repair.^51^ In both groups, serum native free thiol levels decreased directly after surgery. Although an increase in serum free thiols levels is seen 24 hours after the surgery, it did not reach baseline levels. Anesthetics used during surgery also might lead to an increased oxidative stress during surgery, therefore explaining the lower serum free thiol levels directly after surgery.^52,53^ Our study shows that the surgery itself poses a direct burden on the antioxidant capacity of a patient. A patient with already low serum free thiol levels preoperatively is left with only a small antioxidant capacity after surgery, due to the postsurgical decrease in serum free thiol levels. A depleted antioxidant capacity cannot counteract oxidative stress sufficiently and might leave the patient more prone to complications.

### Limitations

Our study has some limitations that warrant recognition. For instance, our study included patients with different kinds of gastrointestinal tumors, which required different types of surgery. Unfortunately, the number of patients was not sufficient to perform a sensitivity analysis according to tumor types. Furthermore, our data did not allow to stratify patients according to lifestyle. Strengths of the present study include, among others, the prospective data collection according to an earlier defined protocol, which allowed us to study the clinical implication and the development of serum free thiol levels prior to and after oncological surgery. To the best of our knowledge, no other study has focused on the association of serum free thiols and postoperative outcomes in patients with gastrointestinal cancer. Therefore, we believe that our study provides novel insights and opportunities for future research.

### Future Perspectives (Clinical Implications)

Because serum free thiol levels can be relatively easy, reliably, and minimally invasively quantified in patients, measurement of these compounds may be of significant interest to clinicians. A patient’s systemic redox status can provide valuable information for the clinician, when considering the patient’s risks and benefits of a certain procedure. As our study is the first describing the association between serum free thiols and complications after oncological surgery, the applicability of these results has to be validated in future studies. Adding the antioxidant capacity of a patient to the established risk profile of a patient might improve counseling of patients and help to identify the most suitable treatment.

Furthermore, there might be possibilities to raise the antioxidant capacity of a patient to prevent complications after treatment. First, prehabilitation can focus on lifestyle factors, such as exercising, reducing BMI, and supporting a smoke-free life. Second, administration of antioxidants before surgery might be an additional treatment option. In some studies, it has already been attempted to raise the antioxidant capacity by supplementing a patient’s diet, but so far results are disappointing. Several studies failed to demonstrate an improvement of the antioxidant capacity after supplementation.^54,55^ One mouse model even showed progression of metastasis in lung cancer after vitamin E supplementation.^56^ In this context, however, it is important to note that exogenous administration of antioxidant substances should be carefully tailored to an individual’s systemic redox status and directed only against pathological overproduction of reactive species. Interference with physiological redox signaling processes may lead to adverse effects. For instance, single-electron oxidation of thiols (forming thiyl radicals) or disturbance of membrane transport signaling processes, which are dependent on disulfides, may occur as adverse effects.^21,57,58^ It is therefore important to understand the complexity of the whole-body redox status better, making it possible to treat patients based on their redox status.

## Conclusions

Our study demonstrates that in patients with gastrointestinal cancer a lower antioxidant capacity, as reflected by serum free thiols, is a risk factor for postsurgical complications and longer in-hospital stay. Moreover, the antioxidant capacity of a patient is affected by the surgery itself, resulting in decreased serum free thiol levels after surgery.

## Supplementary Information

Below is the link to the electronic supplementary material.

Supplementary file1 (DOCX 18 KB)

Supplementary file2 (DOCX 18 KB)
