# Supplementary material for: Oxidative Stress Predicts Post-Surgery Complications in Gastrointestinal Cancer Patients
Source: Ann Surg Oncol. 2022 Feb 17;29(7):4540–7. doi: 10.1245/s10434-022-11412-8 (PMC9174134; doi:10.1245/s10434-022-11412-8)
Supplement: Supplementary file 1 — Supplementary file1 (DOCX 18 KB) [file 10434_2022_11412_MOESM1_ESM.docx]

Supplementary table 1. Biomarkers over time

|  | Before surgery, n=75 | At skin closure, n = 71 | Day 1, n = 71 | Day 2, n= 64 | Δ Before surgery – at skin closure, n = 71 |
| --- | --- | --- | --- | --- | --- |
| Free thiols (g/L) | 279.0 ( ± 50.7) ˟ | 201.2 ( ± 59.0) ˟ | 225.2 ( ± 48.4) ˟ | 197.9 ( ± 42.4) ˟ | -69.9 ( - 94.0 – (-)49.3) |
| CRP (μg/ml) | 9.2 (2.5 – 27.1) | 11.8 (3.5 – 33.4) | 395.8 (317.7 – 665.9) | 681.8 (458.0 -887.3) | 0.0 (-2.7 – 7.4) |
| IL-1β (pg/ml) | 0.3 (0.0 – 11.9) | 0.3 (0.0 – 9.6) | 0.9 (0.0 – 10.7) | 0.8 (0.0 – 14.0) | 0.0 (-3.0 – 0.0) |
| IL-6 (pg/ml) | 1.5 (0.0 – 8.6) | 502.4 (68.6 – 889.1) | 407.3 (215.7 – 897.1) | 239.4 (113.5 – 363.6) | 496.70 (35.1 – 878.5) |
| IL-10 (pg/ml) | 2.82 (0.3 – 6.7) | 22.95 (2.9 – 67.8) | 23.50 (3.3 – 40.0) | 13.90 (2.6 – 24.1) | 11.8 (0.5 – 50.4) |
| IL-12 (pg/ml) | 1.6 (0.9 – 2.8) | 1.50 (0.9 – 3.3) | 1.8 (1.1 – 3.3) | 2.0 ( 1.2 – 3.6) | - 0.1 (-0.6 – 0.6) |
| TNF-α (pg/ml) | 0.5 (0.0 – 7.8) | 1.4 (0.0 – 5.9) | 1.9 (0.0 – 16.0) | 2.6 (0.8 – 28.4) | 0.0 (-0.5 – 0.7) |
| Albumin | 50.8 (47.6 – 59.0)^1^ | 40.2 (33.0 – 49.8)^2^ | 40.4 (31.4 – 46.5)^3^ | 39.62 (32.5 – 42.2) ^2^ | -10.3 (-19.8 – (-) 4.8)^4^ |
|  |  |  |  |  |  |

Data shown as median (25^th^ and 75^th^ percentile) unless specified otherwise, ^1^n = 50, ^2^n = 52, ^3^n = 51, ^4^n = 49 ˟ Data are shown as mean (SD)
