# Supplementary material for: Oxidative Stress Predicts Post-Surgery Complications in Gastrointestinal Cancer Patients
Source: Ann Surg Oncol. 2022 Feb 17;29(7):4540–7. doi: 10.1245/s10434-022-11412-8 (PMC9174134; doi:10.1245/s10434-022-11412-8)
Supplement: Supplementary file 2 — Supplementary file2 (DOCX 18 KB) [file 10434_2022_11412_MOESM2_ESM.docx]

Supplementary table 2. Logistic regression analysis for biomarkers at baseline and length of stay above the median

|  | OR (95% CI) | P-value |
| --- | --- | --- |
| **Free thiol** |  |  |
| First tertile (172.7 – 252.3) | 4.0 (1.3 – 12.9) | 0.01 |
| Second tertile (252.4 – 295.7) | 1.4 (0.4 – 4.2) | 0.60 |
| Third tertile (295.8 – 316.6) | 1 |  |
| **Gender** |  |  |
| Female | 0.6 (0.2 – 1.8) | 0.38 |
| Male | 1 |  |
| **Age** |  |  |
| ≤ 70 | 1.8 (0.8 – 4.7) | 0.18 |
| > 70 | 1 |  |
| **BMI** |  |  |
| Normal BMI (<25) | 1 |  |
| Overweight (25-30) | 1.2 (0.3 – 4.9) | 0.84 |
| Obese (>30) | 2.1 (0.7 – 6.2) | 0.18 |
| **Comorbidity** |  |  |
| ≤ 4 | 1 |  |
| > 4 | 1.2 (0.5 – 3.0) | 0.67 |
| **Tumor stadium** |  |  |
| I - II | 1 |  |
| III - IV | 1.4 (0.4 – 4.7) | 0.62 |
| **Neoadjuvant chemotherapy** |  |  |
| No | 1 |  |
| Yes | 2.4 (1.0 – 6.1) | 0.06 |
| **Type of surgery** |  |  |
| Laparascopy | 1 |  |
| Laparatomy | 0.9 (0.3 – 2.5) | 0.88 |

OR= Odds ratio, CI= Confidence interval, BMI= Body Mass Index
